# Parent-reported experiences of in-laboratory polysomnography in children with neurodevelopmental disorders: A cross-sectional multi-centre study

**DOI:** 10.1007/s11325-025-03333-z

**Published:** 2025-04-24

**Authors:** Ajay Kevat, Dhruv Alwadhi, Andrew Collaro, Anne Bernard, Moya Vandeleur, Karen Waters, Jasneek Chawla

**Affiliations:** 1 Department of Respiratory and Sleep Medicine, Queensland Children’s Hospital, South Brisbane, QLD Australia; 2 Kids Sleep Research Team, Child Health Research Centre, The University of Queensland, 62 Graham Street, South Brisbane, QLD 4101 Australia; 3 Queensland Facility for Advanced Bioinformatics, The University of Queensland, St Lucia, QLD Australia; 4 Department of Respiratory and Sleep Medicine, Royal Children’s Hospital, Parkville, VIC Australia; 5 Murdoch Children’s Research Institute, Parkville, VIC Australia; 6 Department of Sleep Medicine, The Children’s Hospital at Westmead, Sydney, NSW Australia; 7 Discipline of Child and Adolescent Health, Sydney Medical School, University of Sydney, Sydney, NSW Australia

**Keywords:** Paediatric, Polysomnography, Neurodevelopmental disorders, Sleep

## Abstract

**Purpose:**

In-laboratory polysomnography (PSG) is the gold standard test for diagnosing certain paediatric sleep conditions. Children with neurodevelopmental disorders (NDD) often have difficulty tolerating PSG, but parent and patient experiences of PSG for children with NDD have not been thoroughly explored. The study aim was to evaluate the parent-reported experience of in-laboratory PSG undertaken in children with NDD and to identify factors predictive of poorer experience.

**Methods:**

In this cross-sectional multicentre study, parents of 143 children with NDD who underwent in-laboratory PSG completed a customised survey to provide feedback on parent and child worry levels, subjective tolerance and overall experience of PSG, and hypothetical preference between in-laboratory PSG versus an in-home mat-based sleep test. ANOVA, Chi-squared and Kruskal–Wallis tests were used to determine participant factors associated with these outcomes.

**Results:**

On average, parents rated their child’s worry level with respect to undergoing PSG as ‘moderate,’ but their own worry levels lower. Autism spectrum / neuromuscular disorder diagnoses were risk factors for both higher worry score and reporting that sleep during PSG was non-representative of usual sleep at home. Parental preference was for in-home (mat-based) testing, with 57% indicating a preference for this if it wereavailable vs. 7% preferring in-laboratory testing.

**Conclusion:**

Parent/carer reports regarding in-laboratory PSG experiences for their children with NDD suggest the test is associated with child worry and concerns that the sleep is not-representative of usual sleep at home. Consumer preference favours in-home sleep study testing over current in-laboratory diagnostic testing.

**Clinical trial registration:**

This study is part of a larger trial ACTRN12622001544763.

## Introduction

Neurodevelopmental disorders (NDD) encompass a wide range of behavioural and cognitive conditions that emerge during the developmental period, leading to significant difficulties in acquiring and performing intellectual, motor, language, and/or social functions [1]. Children with NDD frequently encounter complex sleep disturbances, such as frequent nocturnal awakenings, insomnia, periodic limb movements, and sleep-disordered breathing (SDB), which can severely impact their health and development [2, 3]. These sleep issues not only affect the child but also cause stress to caregivers and families [4].

The prevalence of SDB in children with NDD is disproportionately high, reaching up to 80%, compared to only 1–6% in the general paediatric population [5]. While various approaches to evaluate suspected SDB have been described, diagnostic polysomnography (PSG) remains the gold standard tool for diagnosing these disorders [6]. While accurate and reliable, PSG tests are expensive, time-consuming and not always well tolerated in NDD cohorts [6–8]. In particular, the application of electrodes and sensors can be distressing for these children with NDD, who often have sensory sensitivities, anxiety, and difficulties with behavioural regulation [9, 10]. When considering that children with NDD may require repeated PSG assessments throughout childhood, these challenges can be an obstacle to their ongoing clinical management.

We previously documented that children with NDDs are three times more likely to have difficulty tolerating PSG leads than their neurotypical peers [8]. That research assessed tolerance of monitoring equipment based on physician and technician report, but did not explore the parent/patient experience. As many aspects of the parent and patient experience of in-laboratory PSG have not been explored for children with NDD, in this multicentre study we explored the experience of children with NDD when undergoing PSG using parent-report of their child’s experience. To our knowledge, no other studies have reported the parents’ perspective of these children’s PSG experience. This research was conducted as part of a broader study examining the diagnostic capability of the Sonomat contactless sleep mat for detection of sleep disordered breathing in children with neurodisability [11]. The following questions were addressed using a customised survey completed by parents/carers immediately after their child with NDD underwent PSG:

- Aim (1) What was the perceived level of parent and child worry, tolerance of PSG set-up and equipment, comparison with typical sleep, parental opinion on in-home sleep mat compared to in-laboratory sleep testing and overall experience of PSG in paediatric NDD cohorts?
- Aim (2) What factors, if any, (particular NDD, age, sex and prior experience of PSG) are predictive of worse outcomes for the issues evaluated in Aim (1)?

We hypothesised that perceived parent and child worry levels would be similar to each other, intolerance of PSG set-up and equipment would be reported in less than one-third of children, and parental preference would be equally divided with respect to a theoretical choice between in-laboratory attended versus in-home sleep testing.

## Methods

### Participants and survey

We conducted a cross-sectional study of 143 children aged 0–18 years with NDD who underwent in-laboratory polysomnography (PSG) at the Queensland Children's Hospital (QCH), the Children's Hospital at Westmead (CHW), and the Royal Children's Hospital (RCH) between January 12, 2021 and May 31, 2024, using a custom-designed paper-based questionnaire (Appendix). Human research ethics committee approval was obtained (HREC reference: HREC/22/QCHQ/89813).

Participants comprised parents of children with a clinician-diagnosed NDD. Children without a confirmed diagnosis of NDD or with absent survey data were excluded; partially completed surveys were included.

### In-laboratory polysomnography

In-laboratory PSG studies were conducted for clinical indications in three Australian tertiary paediatric sleep laboratories following American Academy of Sleep Medicine (AASM) recommendations [12]. Recording included electroencephalography (EEG), electro-oculography (EOG), sub-mental and diaphragmatic electromyogram (EMG), thoraco-abdominal respiratory inductance plethysmography bands, nasal pressure transducer and oronasal thermistor. Cardiorespiratory variables (heart rate, electrocardiogram (ECG), respiratory rate, pulse oximetry and carbon dioxide levels) were also recorded and audio-visual recordings and body position were obtained. Studies were conducted for a maximum of 10 h overnight. Multiple limb leads were only applied if specifically requested by the referring sleep specialist. While children may be shown PSG equipment prior to the study and relevant information is always provided to carers, there is no standardised desensitization protocol for children undergoing PSG in any of the participating tertiary centres. Immediately following completion of PSG, parents were asked to complete the study-specific 9-item (Appendix). After completion of the survey, responses with participant demographic information and their NDD condition were collated using RedCap® and exported to a Microsoft Excel® electronic spreadsheet for data analysis.

### Data analysis

NDD diagnoses were classified into thirteen subgroups: craniofacial disorders, structural CNS malformations, neuromuscular conditions, global developmental delay (GDD)/intellectual disability (ID), cerebral palsy, attention deficit hyperactivity disorder (ADHD), foetal alcohol syndrome, Tourette syndrome, autism spectrum disorder (ASD), Trisomy 21, Prader-Willi Syndrome (PWS) and other genetic disorders. Participants with multiple diagnoses were included in multiple subgroups corresponding to their diagnoses.

The Shapiro–Wilk test was used to determine whether continuous data were normally distributed. Data with a normal distribution were reported as mean and standard deviation. Non-normally distributed data was reported as median and interquartile range values. For ordinal responses, such as child worry and parent worry (1–10), linear regression was used to compare the likelihood of successful PSG setup with the presence of confounders, with Analysis of Variance (ANOVA) used to analyse differences across groups. To analyse the association between categorical responses such as ease of setup, comparison with typical sleep and overall experience, Chi-squared tests were used.

In order to account for non-normal data distribution, Kruskal–Wallis testing was used to evaluate the relationship between age (a continuous variable) and categorical variables, such as equipment retention and typical sleep. Data was processed using the R statistical package (R Foundation for Statistical Computing, Vienna, Austria), with one-tailed p-values less than 0.05 considered statistically significant. Figures were generated using GraphPad Prism (Graphpad Software, Boston, United States of America).

## Results

### Participant characteristics

**Fig. 1:**
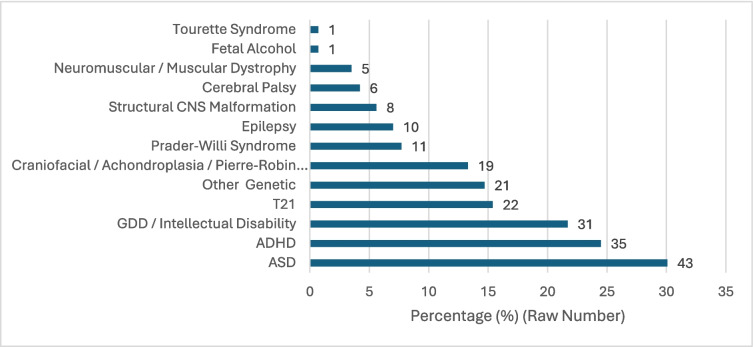
Neurodevelopmental disorder subgroups. Percentages given on horizontal axis, with raw numbers given beside each bar

A total of 161 children were consented for the study, with 18 excluded due to failure to return the survey form, resulting in a final sample size of 143 participants. The median age of participants in the study was 10.9 years (IQR = 6.5–14.3); 44% of the participants were female. The majority of participants were from QCH in Brisbane (72%), followed by RCH in Melbourne (22%) and CHW in Sydney (6%). Further demographic and anthropometric characteristics of the study group are shown in Table 1. The most common diagnoses for the cohort were Autism Spectrum Disorder (ASD) (30%), Attention Deficient Hyperactivity Disorder (ADHD) (24%) and Global Developmental Disorder (GDD)/Intellectual Disability (22%). Just over half (51%) of participants had undergone previous diagnostic in-laboratory PSG (Fig. 1).

**Table 1:** Participant demographic and anthropometric characteristics

Variable	Overall
n	143
Age (median, interquartile range)	10.85 (6.45–14.3)
Sex	
Male (n, %)	80 (66%)
Female (n, %)	63 (44%)
Anthropometric Measurements	
Height (cm; median, interquartile range)	137. (114.2–156.7)
Weight (kg; median, interquartile range)	38.9 (21.8–58.7)
BMI (median, interquartile range)	19.6 (16.9–25.5)
Prior study	
Yes (n, %)	73 (51%)
No (n, %)	64 (45%)
Don’t know/Unanswered	6 (4%)
Clinical Site	
Queensland Children’s Hospital (n, %)	103 (72%)
Royal Children’s Hospital (n, %)	31 (22%)
Westmead Children’s Hospital (n, %)	9 (6%)

### Parent and child reported worry

**Fig. 2:**
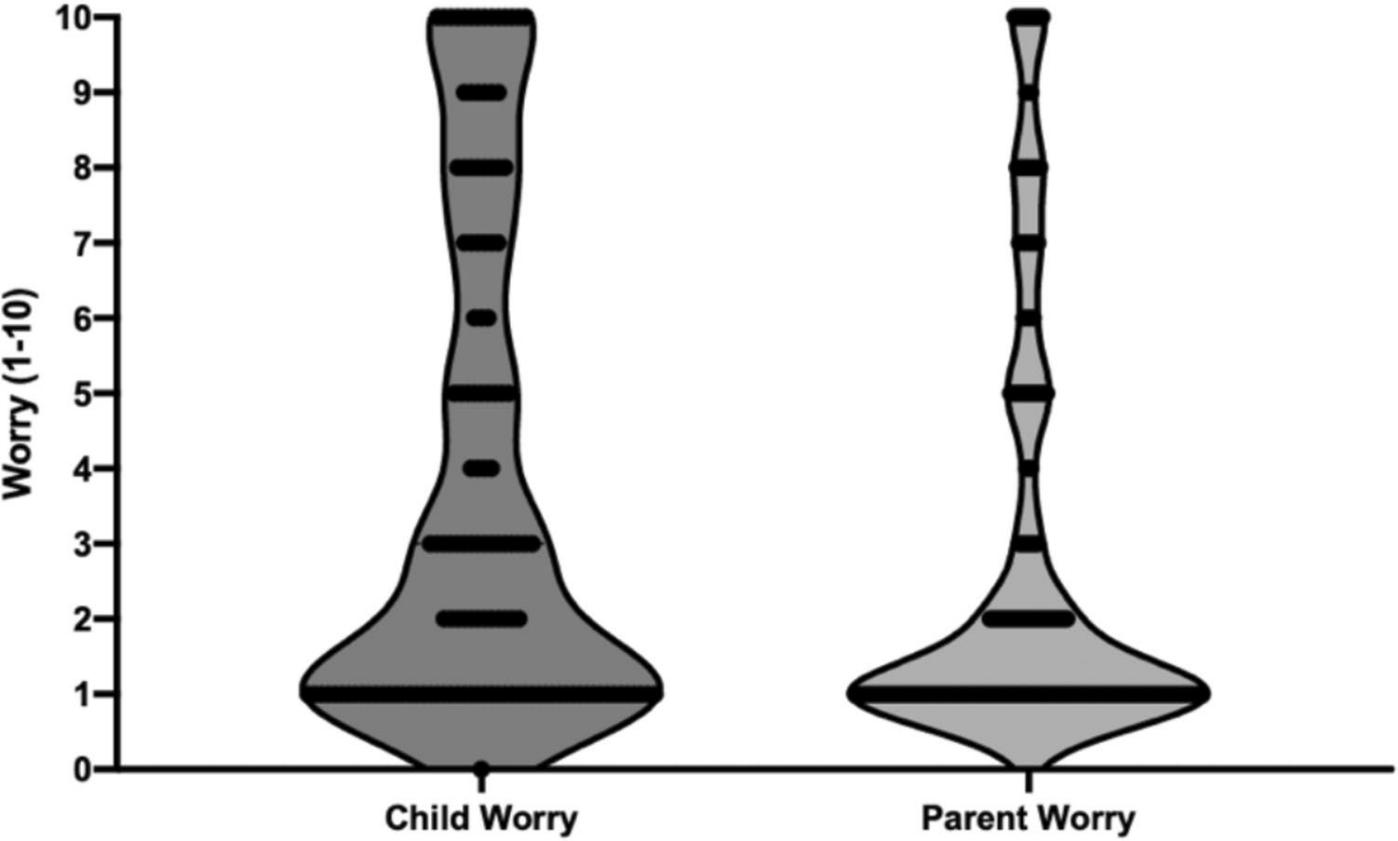
Violin plots representing the distribution of overall cohort worry scores (rated on a scale of 1–10) reported by children and their parents during in-laboratory polysomnography. The left plot illustrates child-reported worry, whilst the right plot shows parent-reported worry. Plot width reflects the distribution and frequency of worry levels within each group

**Fig. 3:**
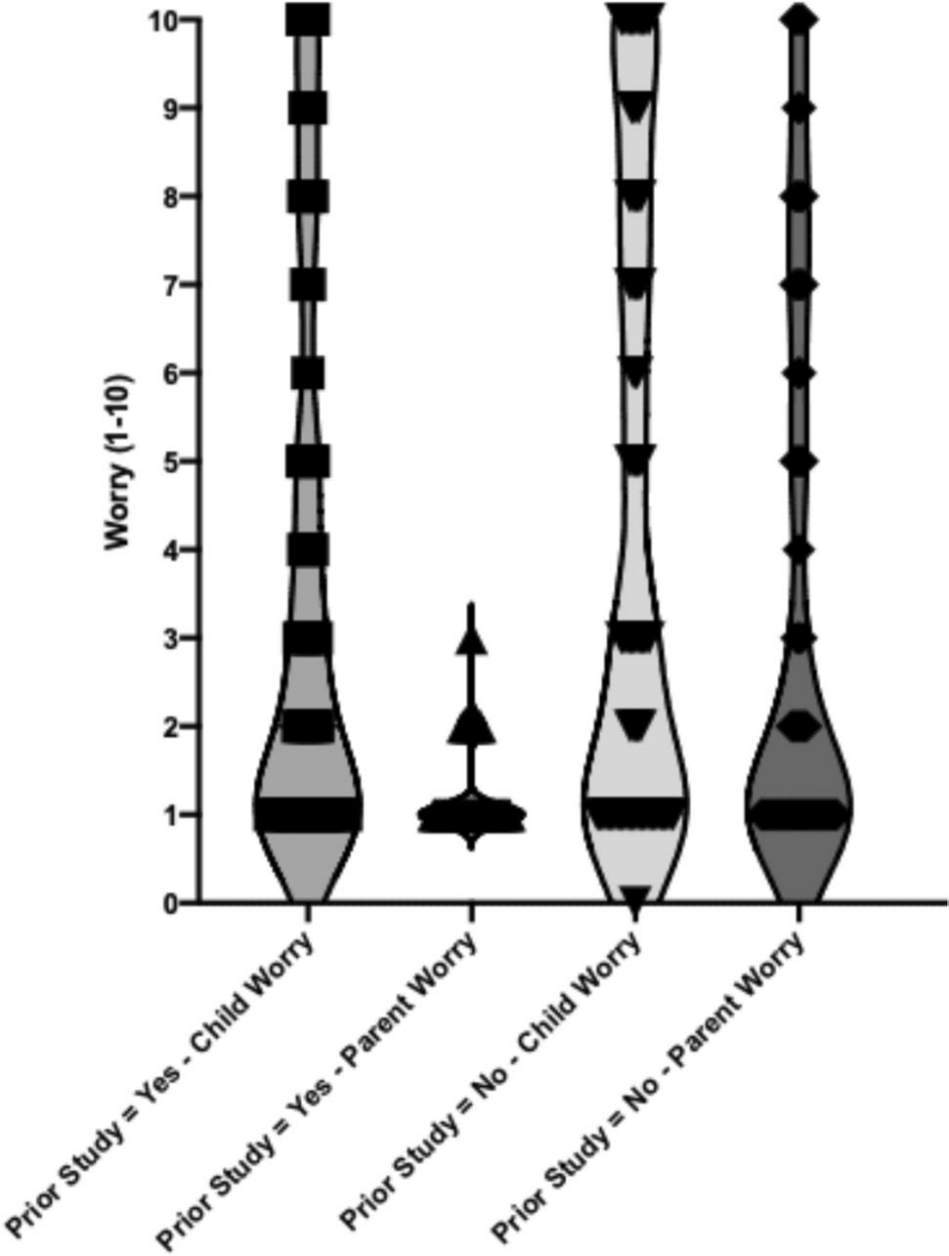
Violin plots displaying worry scores (rated on a scale of 1–10) from both children and parents, categorised by whether they had prior experience with PSG (Polysomnography). The first two plots represent child and parent worry scores when there was prior PSG experience, while the last two plots represent the worry scores when there was no prior experience. Plot width reflects the distribution and frequency of worry levels within each group

From parent-report, children experienced higher levels of worry than their parents during the PSG process, with a median child worry score on a 1–10 point Likert scale of 3 [IQR: 1–7] compared to median parent worry score of 1 [IQR: 1–3.5]. ANOVA analysis indicated two diagnostic labels were significantly associated with an increase in child-reported worry, namely: Neuromuscular/Muscular Dystrophy (*p* < 0.01) and ASD (*p* < 0.01). Similarly, ANOVA analysis found significant increases of parent-worry for children with Neuromuscular/Muscular Dystrophy (*p* = 0.02) and ASD (*p* = 0.02). Additionally, parents of children who underwent testing at CHW and those without prior PSG experience reported higher worry (*p* < 0.01 and *p* = 0.03 respectively) (Figs. 2 and 3).

### Tolerance of PSG set-up and monitoring equipment

Overall, most parents (36%) reported the setup was"Easy"for their child, although a notable portion (22%) reported this as “Hard,” with a further 6% rating it"Very Hard."Chi-squared testing revealed a significant association between perceived setup difficulty and ASD (*p* = 0.02).

The majority of parents (76%) reported that monitoring leads were successfully maintained for the duration of the study. When examining association with NDD category, Chi-squared tests found significant associations for Cerebral Palsy (*p* = 0.05) and GDD/Intellectual Disability (*p* = 0.04). Children with Cerebral Palsy struggled more with monitoring, and 40% experiencing issues compared to 8% for those without this condition. Similarly, 21% of children with GDD/Intellectual Disability had monitoring problems, compared to 6% of children without this diagnosis.

### Sleep similarity

Participants were divided on whether they felt their child's sleep in the laboratory mirrored their typical sleep at home, with 37% reporting similarity and 36% indicating dissimilarity, with a further 27% being unsure or providing no answer. Chi-squared tests found significant associations between perceived atypical sleep for children with ASD and neuromuscular disease (*p* = 0.01 for both). Of the families with ASD, only 24% felt their child's sleep during the PSG was similar to sleep at home, compared to 50% for non-ASD parents. For children with a neuromuscular disorder, none of the parents/carers reported similar sleep during PSG compared to sleep at home.

### Overall experience and future study preference

**Fig. 4:**
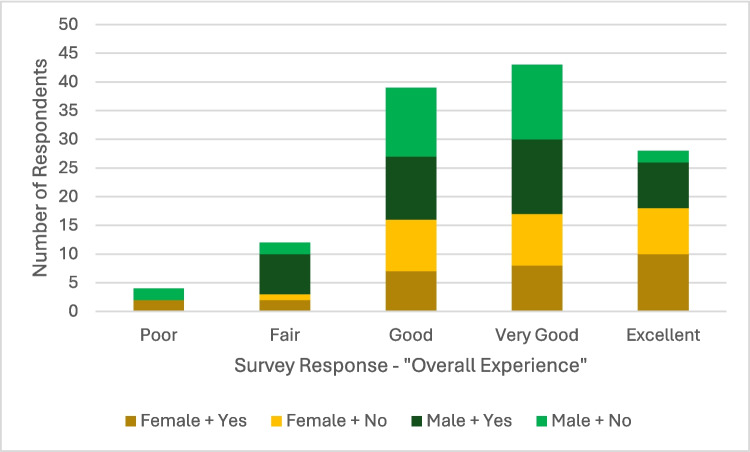
Stacked bar chart representing the overall experience ratings of in-laboratory polysomnography (PSG) reported by parents, grouped by child’s sex (male/female) and prior PSG experience (Yes/No). Experience ratings are categorised from"Poor"to"Excellent,"with each bar displaying the number of respondents. Colours indicate the breakdown between males and females, as well as those with and without prior PSG experience

The majority of parents/carers (57%) rated the overall PSG experience positively, with 30% rating it"Very Good"and 27% rating it"Good"with Chi-squared test excluding age, NDD diagnosis, sex or survey centre as influencing the reported experience: Kruskal–Wallis test confirmed that age did not affect overall experience (*p* = 0.35). A final survey item asked parents/carers about their theoretical preference between a hospital-based sleep study and an in-home study using the Sonomat sleep mat for their child, in which the majority (59%) indicated a preference for the home-based study, while only 7% preferred the in-laboratory study (Fig. 4).

## Discussion

This is the first study to assess the parent-reported experience of in-laboratory PSG specifically in children with NDD and highlights the challenges for this population. Several key findings have important clinical implications.

Firstly, in keeping with reports from others who examined the experiences of children (although not specifically those with NDD) undergoing PSG [13], our study found more than a third of parents/carers felt that their child’s sleep on the night of the PSG was not typical of sleep in their home environment. This may be due to an increased propensity for sleep fragmentation and night waking in younger children [14] which parents may become aware of on the night of the PSG when they are required to sleep in the same room as the child, as well as due to the ‘first-night effect’, a well-documented phenomenon of poorer sleep experienced on the first night in a new location, observed in both children and adults [15]. Atypical sleep during a diagnostic test suggests that PSG may not accurately capture usual sleep architecture and sleep stages, which could lead to underestimating the severity of SDB and suboptimal treatment decisions. A retrospective study of 200 participants undertaken over a decade ago found that more than a third of respondents felt their child’s breathing on the night of the PSG did not reflect that usually seen at home [16]. In-home testing is likely to result in more typical patterns of sleep, given the usual sleep environment, particularly for children with NDD in whom small changes in routine may be difficult.

Secondly, we found that prior experience of PSG resulted in reduced worry amongst children and their parents. The higher reported parent and child worry regarding first-time PSG demonstrated in our study is similar to patterns of greater reported anxiety during other first-time procedures such as radiological imaging [17] and surgeries [18]. A common thread across these studies is reference to desensitisation protocols as an avenue for reducing anxiety in participants. Research with a particular focus on PSG desensitisation has demonstrated that after an average of approximately four sessions involving home practice and a tour of the sleep laboratory, 19 out of 23 child participants successfully completed PSG without sedation [19]. PSG desensitisation strategies may therefore be of benefit to some children. Evaluating differing PSG desensitisation approaches for children with NDD is an important area for future research.

A previously unreported finding from our study is the higher reported parent and child worry scores and increased likelihood of atypical sleep observed in the sub-populations of children with ASD and/or neuromuscular disease. We speculate that reasons for this could include children with ASD having propensity for sensory sensitivities being triggered by PSG monitoring leads, comorbid anxiety disorders, difficulty tolerating changes to routines, and children with neuromuscular weakness having decreased ability to co-ordinate motor withdrawal from noxious stimuli, perhaps increasing fear of PSG-related procedures [20, 21]. It should also be noted that there is a well-recognised overlap between neuromuscular disease and ASD – particularly in children with Duchenne Muscular Dystrophy [22]. In some cases, worry expressed by parents and children may point to a feedback loop where the discomfort of the child increases the anxiety of the parent and vice versa, thereby making experiences for both worse overall [23]. Limited desensitisation sessions alone may not be sufficient to address this problem, although extensive parental education and emotional support could assist in breaking this cycle.

A final novel finding from our study is the clear preference reported by parents for an in-home mat-based diagnostic option compared to only 7% of families favouring the in-laboratory evaluation. We speculate that this is likely motivated by the distress and anxiety brought about by additional hospital visits, the burden of care associated with transporting children with NDD, and the impact of a new test/medical environment. It should be noted the questionnaire specifically enquired about home sleep mat-based testing, rather than other more traditional forms of home sleep apnoea tests (HSATs), such as Level 2 HSATs. Important future work should explore in greater detail the factors driving parental preference for home-based testing, with qualitative studies providing the option to examine families’ experiences and attitudes towards in-laboratory and in-home testing of various types.

This study has a number of limitations. Firstly, while we assessed the experiences of parents and other caregivers, we did not evaluate the experiences of children directly. Whilst some children with NDD may be too young or unable to effectively communicate their feelings regarding PSG experience, many children with neurodevelopmental conditions may be able to reliably provide feedback, and evaluating this in future research is strongly recommended. Second, the questionnaires’ PSG equipment set-up and overnight tolerance report was intrinsically subjective because it relied on parent report rather than objective assessment. By accompanying their child for the study and sleeping in the same room, parents may become more acutely aware of any sleep disturbances, potentially heightening the degree of perceived distress/worsened sleep quality compared to what might be perceived in the home environment. Previous research has assessed PSG set-up and monitoring lead tolerance in children with NDD more objectively [8]. These reports may or may not align with parental perception; as an example, caregiver reports of sleep in children with ASD frequently differed from actigraphy data [24]. Caregiver reports are subject to recall bias. A further limitation is the potential variability in the PSG setup across sites. Minor differences in the way technicians demonstrated or used the equipment, the amount of support provided, or environmental factors such as the arrangement, and room ambience could impact the child’s experience and tolerance. Additionally, although we aimed to examine PSG experiences from three sites, the number of patients from QCH (72% of participants), was disproportionately large, making it difficult to draw conclusions about differences between locations. A final limitation is the lack of a direct comparison group of neurotypical children.

## Conclusion

In-laboratory PSG is a challenging test for children with NDD, where there is considerable risk of the child experiencing worry, suboptimal sleep during the procedure, and difficulty tolerating the application of the monitoring equipment. We were able to identify subgroups at greater risk including those with younger age, and a diagnosis of neuromuscular or autism spectrum disorder. Familiarity with the procedure through past exposure decreased parent worry, suggesting that PSG desensitisation may help some children. Finally, consumer preference was for in-home testing if possible. We conclude that progressing alternative methods of comprehensive sleep evaluation [25, 26] and translating novel non-invasive measurement technologies [27] for in-home use should be a priority and would confer particular benefits for children with NDD.

## Data Availability

The data that support the findings of this study may be made available in deidentified format from the corresponding author upon reasonable request.

## Appendix

Sleep Study Questionnaire: Sonomat in Neurodisability (ND) – In Lab Hospital Sleep Study (PSG). We would be very grateful if you could answer the questions below about the in-lab sleep study your child had last night.

1. Has your child had a sleep study before the one last night? (Yes/No/Don’t Know).
2. How worried was YOUR CHILD about doing a hospital sleep study? (Please circle your answer: 1 = not worried at all, 10 = very worried) [Scale: 1–10].
3. How worried were YOU about your child doing a hospital sleep study? (Please circle your answer: 1 = not worried at all, 10 = very worried) [Scale: 1–10].
4. Overall, how easy did your child find the sleep study set up? (Very Easy/Easy/Neither/Hard/Very Hard).
5. Do you think your child had a typical sleep during the sleep study? (Yes/No/Don’t Know).
6. Did your child manage to keep the sleep study monitoring on most of the night? (Yes/No/Don’t Know).
7. Overall, how would you rate your experience of the sleep study? (Poor/Fair/Good/Very Good/Excellent).
8. Any other comments about the Sleep Study? [Open-ended response].
9. If you had a choice between a hospital sleep study and using the Sonomat sleep mat at home, which would you prefer? (Hospital sleep study/Sonomat Sleep Mat/Either/Don’t know).

Thank you for your participation. CHQ Carer Questionnaire, V2.0 dated 09 February 2023.

From these, all survey items excluding item 8 (i.e. 1–7, and 9) were used in analysis.

## Declarations

### Permission to reproduce material from other sources

N/A.
